# Case Report: A complex case of an adolescent female with comorbid borderline personality disorder and autism spectrum disorder

**DOI:** 10.3389/fpsyt.2025.1681412

**Published:** 2025-09-22

**Authors:** Sara Močnik, Hojka Gregorič Kumperščak, Ajda Demšar

**Affiliations:** ^1^ Unit for Paediatric and Adolescent Psychiatry, Division of Paediatrics, University Medical Centre Maribor, Maribor, Slovenia; ^2^ Laboratory for Digital Signal Processing, Faculty of Electrical Engineering and Computer Science, University of Maribor, Maribor, Slovenia; ^3^ Department of Psychiatry, Faculty of Medicine, University of Maribor, Maribor, Slovenia; ^4^ Center for Hearing and Speech Maribor, Maribor, Slovenia

**Keywords:** case report1, Borderline personality disorder2, autism3, comorbidity4, adolescent5

## Abstract

Diagnosing borderline personality disorder (BPD) and autism spectrum disorder (ASD) in adolescent females presents significant challenges due to the overlap in their symptomatology. Both conditions share features such as emotional dysregulation, impulsivity, and difficulties in interpersonal relationships, making differentiation crucial yet difficult. This case report examines an adolescent female with co-occurring BPD and ASD, emphasizing the complexities of distinguishing between the two. It explores the role of developmental history, behavioral patterns, and neurobiological factors in forming a precise diagnosis. Additionally, the report highlights the impact of comorbid conditions like depression and anxiety, which frequently accompany both BPD and ASD, further complicating the diagnostic process. By focusing on this case, we underscore the importance of a thorough, multidimensional diagnostic approach to ensure accurate identification and treatment. The case serves as a reminder of the need for heightened awareness of how BPD and ASD can present in females and advocates for more nuanced diagnostic tools and tailored interventions to improve clinical outcomes for this population.

## 1 Introduction

Borderline personality disorder (BPD) is a complex condition characterized by emotional dysregulation, impulsivity, unstable self-image, and interpersonal difficulties. Often comorbid with depression, anxiety, and substance abuse, BPD is difficult to diagnose in adolescents due to symptom overlap and a lack of a clear diagnostic model (1, 2). Although lifetime prevalence is 2.7%, rates are significantly higher in clinical settings, especially among females, with early traits often emerging in childhood. Early intervention is crucial (1, 3), yet stigma and misdiagnosis often delay timely and proper treatment (1, 4–6).

Autism spectrum disorder (ASD) involves deficits in social communication and restricted, repetitive behaviors (7). Affecting about 1 in 130 children globally (8) and 1 in 31 in the United States (9), ASD often co-occurs with intellectual disability and psychiatric disorders. Females are frequently underdiagnosed due to camouflaging behaviors and comorbid conditions like eating disorders and PTSD (10, 11).

BPD and ASD can share features such as emotional dysregulation, impulsivity, self-injury, social difficulties, identity confusion, interpersonal sensitivity, and cognitive rigidity, among others (12, 13, 14).^,,^ Up to 50% of individuals with ASD meet criteria for a personality disorder, and BPD is often misdiagnosed, especially in females, where emotional lability and self-harm may overshadow autistic traits (11). High autistic traits in BPD are also linked to increased suicidality and self-injury (12).

This case report describes the diagnostic and treatment course of an adolescent female with comorbid BPD and ASD, complicated by a complex clinical picture and strained family dinamics. The case highlights several challenges: difficulties in obtaining a reliable developmental history due to strained family dynamics, limited utility of self-report instruments influenced by emotional dysregulation, and diagnostic uncertainty stemming from overlapping symptoms. The patient’s clinical presentation and longitudinal course illustrate the importance of nuanced assessment and ongoing diagnostic reevaluation. Ultimately, this case underscores the clinical utility of individualized interventions when managing co-occurring BPD and ASD in adolescent females.

## 2 Case description

A 17-year-old girl entered our outpatient psychiatric care at age 16 during a period of escalating emotional distress, self-harm, and suicidal ideation. She had no prior psychiatric diagnosis and had dropped out of school.

### 2.1 Patient information

Her first contact with medical services followed a suicide threat, resulting in a brief ER visit and a short benzodiazepine prescription without psychiatric follow-up. She later received weekly support from a non-clinical psychologist for a year before being referred to child and adolescent psychiatry.

Living with her mother, stepfather, and younger half-siblings until age 16, she moved to her maternal grandmother’s home due to ongoing family conflict. Her parents divorced when she was four; her father, who struggled with alcohol misuse, was largely absent. Her mother, emotionally unstable with alcohol dependence, played a central role in her early life. Family psychiatric history was explored. While both parents had histories of alcohol misuse and emotional instability, no clear family history of bipolar disorder or other major mood disorders was identified.

Early neurodevelopment was marked by advanced literacy and numeracy skills, sensory sensitivities, and prolonged pacifier use. She was described as an easy infant but became emotionally reactive and demanding in early childhood.

She is overweight, experiences evening binge eating, and has irregular menstrual cycles. No further somatic concerns were noted.

Academically, she performed well in elementary school despite poor concentration and minimal effort. After a negative high school experience, she developed school avoidance but showed interest in evening school or employment.

The patient formed intense online relationships, often with transgender peers, characterized by rapid attachment, idealization, and emotional investment. However, these relationships were abruptly ended, leading to distress, crying, and dissociative symptoms.

She reported significant adverse childhood experiences, including emotional neglect, maternal alcohol use, domestic violence, and chaotic home environments. She took on caregiving roles for her siblings, with frequent verbal and physical aggression between her and her caregivers. While she briefly mentioned an unwanted sexual experience with peers, she declined further discussion.

She presented with chronic low mood, fatigue, suicidality, anxiety, panic attacks, emotional dysregulation, impulsivity, self-harm, and a complex differential diagnosis involving BPD, ASD, OCD, ADHD, and trauma-related symptoms. Conduct disorder was not clearly present, although aggression occurred during dysregulated states.

### 2.2 Initial clinical assessment

Following a GP referral for an unspecified mood disorder (F39, ICD-10), the patient was first evaluated by a child and adolescent psychiatrist in the emergency setting. She presented with low mood, anxiety, a complex family environment, and limited peer relationships, leading to a referral for ongoing care in the outpatient service.

At the initial outpatient assessment, she described depressive episodes marked by social withdrawal, poor hygiene, and a cyclical avoidance pattern—feeling unable to clean herself if her room was dirty and vice versa. Her affective states fluctuated, including crying spells, irritability, and brief periods of increased energy.

Self-harming behavior had ceased after moving in with her grandmother, though intrusive urges remained. Past self-injury included burns from lighters, wax, and plastic. She expressed long-standing interests in astronomy, Greek mythology, religion, psychology, and true-crime documentaries.

Obsessive-compulsive traits were noted, including rigid routines, such as washing her hair on specific days, drinking water only from a particular glass and jug, and preferring even numbers (excluding 6). She became distressed if numbers did not end in 0 or 5, bit her nails until they bled if uneven, and repeatedly folded laundry to meet her standard of perfection. She also experienced intrusive thoughts, which she managed by writing them down.

Attention and concentration difficulties had contributed to academic stress and avoidance. Despite being reflective and cooperative, her speech was often circumstantial, requiring multiple sessions to gather a coherent symptom profile.

### 2.3 Diagnostic challenges

This case highlights the diagnostic challenges in adolescent psychiatry, where rapid emotional and cognitive changes complicate the differential diagnostic process (15). Initially, the patient’s symptoms seemed to align with mood or anxiety disorders, but further assessment revealed broader issues in emotional regulation, identity, and interpersonal functioning consistent with BPD (16). An important factor to consider was the possibility of bipolar disorder, given the presence of mood symptoms, shifts in emotional state, and only partial response to medication (17).

A key challenge was differentiating depressive episodes linked to BPD from a separate depressive disorder. While depressive symptoms are common in BPD, distinguishing it from Major Depressive Disorder (MDD) is crucial (18, 16). The patient’s emotional reactions, often triggered by interpersonal stress, along with identity disturbance and fear of abandonment, are core features of BPD and further complicate the diagnosis (19).

Strained family relationships, particularly with her mother, contributed to emotional instability. Family dynamics, childhood trauma, and both parent’s substance abuse likely exacerbated symptoms. BPD often emerges during adolescence and is influenced by early trauma, insecure attachment, and family dysfunction (20, 21, 22). (23).

The patient’s preference for rigid routines and sensory sensitivities raised the possibility of ASD (7), but it was unclear whether these traits were neurodevelopmental or secondary to emotional distress (10). Individuals with BPD often develop rigid routines as coping mechanisms (24, 25, 12). (11). Additionally, other conditions such as OCD, anxiety disorders, trauma-related disorders (e.g., PTSD or CPTSD), eating disorders, and intellectual disabilities should be considered in the differential diagnosis. Obsessive-compulsive traits, like compulsive nail-biting and specific number preferences, raised concerns about OCD, but could also be maladaptive emotional regulation strategies often seen in BPD (26). Furthermore, distinguishing between OCD and the ritualistic behaviors seen in ASD can be difficult, as both share features like rigidity and restricted interests (27). Additionally, the patient exhibited binge eating, a behavior common in BPD, often linked to emotional dysregulation and fear of abandonment, rather than impulsivity alone (28). It is often triggered by feelings of emptiness and intensified negative emotions post-binge (29).

Attention difficulties and impulsivity suggested potential ADHD, which often coexists with BPD. While both conditions share emotional dysregulation and impulsivity, in BPD, these traits are more stress-related, while in ADHD, they are more constant (30).

Given the patient’s trauma history, Complex PTSD (CPTSD) had to be considered due to overlapping features with BPD – particularly emotional dysregulation, interpersonal difficulties, a negative self-concept, and impulsivity (31, 32, 25).^,,^

### 2.4 Differential diagnosis process

Given the complexity of the patient’s presentation, the differential diagnosis required a structured, multi-method approach (15, 25, 11).^,,^ This included a thorough review of clinical history, structured interviews, standardized psychological testing, and consultation within a multidisciplinary team. The working hypotheses considered BPD (2), ASD (7), OCD (27), bipolar disorder (17), social anxiety (10), and trauma-related disorders (20).

Psychometric testing was conducted by a clinical psychologist using a battery of standardized instruments, including:

- Cognitive and executive function measures: *Wechsler Adult Intelligence Scale–Fourth Edition (WAIS-IV,* 33), *Tower of London Test (TOL,* 34), *Conners Continuous Performance Test–Third Edition (CPT-3,* 35)
- Personality and emotional functioning assessments: *Personality Assessment Inventory–Adolescent Form (PAI-A,* 36), *Operationalized Psychodynamic Diagnosis–Child and Adolescent Form, Second Edition (OPD-CA2-SQ,* 37)
- Trauma and behavioral assessments: *Trauma Symptom Checklist for Children (TSCC,* 38), *Child Behavior Checklist (CBCL 6–18,* 39), *Youth Self-Report (YSR 11–18,* 40)
- Autism-specific evaluations: *Autism Diagnostic Observation Schedule–Second Edition (ADOS-2,* 41), *Adaptive Behavior Assessment System–Third Edition (ABAS-III,* 42)
- Executive functioning ratings: *Behavior Rating Inventory of Executive Function–Second Edition (BRIEF-2,* 43)

The differential diagnosis process focused on evaluating the overlapping and distinct features of these conditions to guide further assessment and ensure a thorough understanding of the patient’s clinical profile.

### 2.5 Final diagnosis

The patient was initially diagnosed with BPD, as her clinical presentation clearly met all nine DSM-5 criteria (44). She displayed affective instability, chronic feelings of emptiness, impulsivity, intense and unstable interpersonal relationships, identity disturbance, inappropriate anger, transient dissociation, frantic efforts to avoid abandonment, and recurrent self-injury. These symptoms were pervasive and significantly interfered with her daily functioning. She experienced significant dissociative symptoms, often describing a sense of derealization and detachment, “like being an avatar in a video game,” accompanied by episodes of lost time during stress or emotional arousal. Early in treatment, these states occurred daily, sometimes multiple times a day, disrupting her sense of continuity and agency, but with therapy they gradually decreased to weekly episodes before resolving. While her symptoms did not meet full criteria for Dissociative Identity Disorder (DID), the presence of time loss and identity disturbance underscored the severity of her trauma-related dissociation and required careful monitoring for DID-like features (45).

However, as the therapeutic process unfolded, it became increasingly clear that her difficulties extended beyond emotional dysregulation alone. She showed longstanding challenges in social communication, marked rigidity, sensory sensitivities, and restricted interests—features not fully accounted for by the BPD diagnosis. These traits had likely gone unnoticed earlier, in part due to masking behaviors and the dominance of mood-related symptoms.

The patient received a score of 13 on the ADOS-2, indicating the presence of clinically significant autism-related characteristics (41). On the ABAS-3, both informant and self-report ratings indicated a generally average level of adaptive functioning, but with meaningful discrepancies across domains. The patient’s grandmother rated her General Adaptive Composite (GAC) at 93 (47th percentile), with scores reflecting low average Conceptual skills (28; 30th percentile), average Social skills (20; 50th percentile), and above average Practical skills (45; 70th percentile). In contrast, the patient’s self-assessment yielded a slightly higher GAC of 99 (53rd percentile), with average Conceptual skills (34; 61st percentile), below average Social skills (18; 19th percentile), and similarly above average Practical skills (47; 66th percentile, 42). The significant discrepancy in perceived social functioning—where the patient reports more pronounced difficulties than observed by the grandmother—aligns with features commonly seen in ASD, such as internalized social struggle despite outwardly adequate behavior. This pattern may reflect camouflaging, a socially motivated strategy in which autistic individuals mask their traits to fit into a predominantly non-autistic world. While this can help them gain acceptance, it is also linked to negative psychosocial outcomes, including low self-esteem, identity confusion, and emotional exhaustion (46).

Although trauma and anxiety-related symptoms were present, they did not meet full diagnostic criteria (44) and were considered contributing factors rather than primary diagnoses.

**Figure 1:**
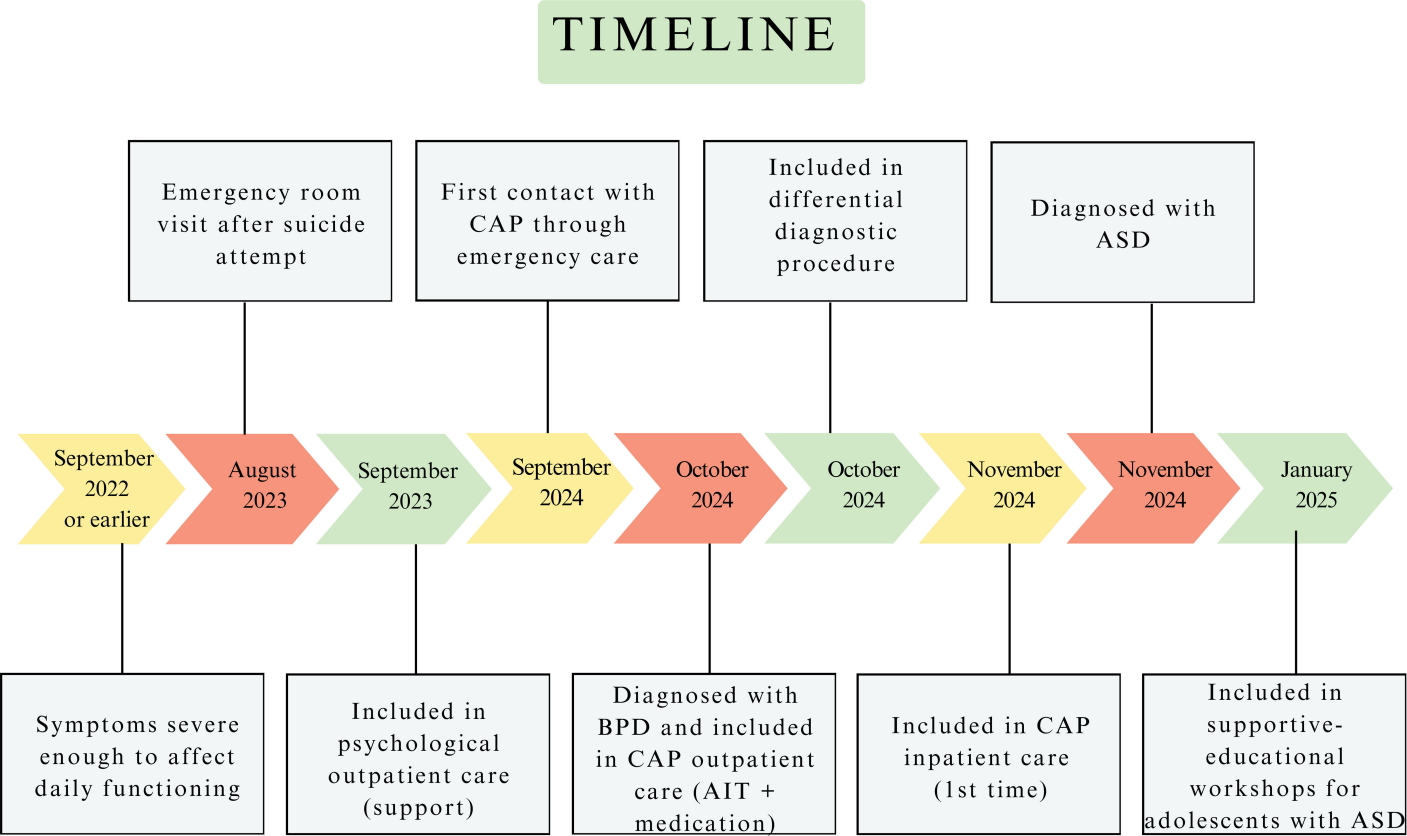
Chronological representation of diagnostic process.

Ultimately, the patient received a dual diagnosis of BPD and ASD. This recognition enabled the development of a more accurate and individualized treatment plan that accounted for both emotional instability and neurodevelopmental challenges. **Figure 1** shows a chronological representation of her diagnostic process.

## 3 Case management

### 3.1 Therapeutic intervention

Pharmacological treatment played an important role in managing the patient’s symptoms. While no medications are specifically approved or recommended for BPD or ASD, antipsychotics are often used for managing mood and dissociative symptoms in BPD (1, 47). SSRIs like fluoxetine are first-line treatments for depression, anxiety, and OCD in youth (48, 49). Fluoxetine was initiated at 20 mg/day during emergency care for depressive symptoms and OCD-like behaviors, with the dose increased to 40 mg/day after limited response. This adjustment resulted in some improvement in anxiety-related behaviors. While subsequent evaluation clarified that depression was not the primary diagnosis, fluoxetine was continued for its partial therapeutic benefit, favorable tolerability, absence of adverse effects, and a low withdrawal risk at discontinuation (48). It is considered safe for adolescents, as it has a lower risk of inducing suicidal ideation compared to other antidepressants (50).

Due to persistent symptoms, particularly involving emotional dysregulation and dissociation, the second-generation antipsychotic brexpiprazole was introduced at 2 mg/day. Its pharmacological profile, including partial agonism at dopamine D2 and serotonin 5HT1a receptors and antagonism at 5HT7, supported improvements in emotional control, anxiety, cognitive rigidity, and dissociative states. Brexpiprazole also offers a lower risk of extrapyramidal symptoms and hyperprolactinemia compared to alternatives like aripiprazole (51). Its relatively lower metabolic risk profile made it an appropriate choice, given the patient’s overweight status (51). While evidence on brexpiprazole use in adolescents with BPD remains limited, its introduction in this case was guided by a detailed clinical rationale and resulted in notable symptom improvement.

In addition to pharmacotherapy, psychotherapy played a key role in long-term treatment. The patient was enrolled in Adolescent Identity Treatment (AIT), a structured intervention targeting self-stabilization, relational functioning, and emotion regulation. AIT integrates psychoeducation, family engagement, and a home plan, addressing identity disturbances and fostering self-esteem (52, 53). The patient participated in psychoeducational workshops for adolescents with ASD, focused on social skills and emotion regulation. Led by a team of specialists—including a psychologist, occupational therapist, speech therapist, and special education professor—the sessions combined theory and practical tools to support daily functioning. Topics included sensory processing, self-care, learning strategies, and mental health. This, along with biweekly outpatient visits that included continuous AIT and pharmacotherapy, led to partial but meaningful improvements in her daily functioning and interpersonal behavior.

### 3.2 Follow-up and outcomes

The patient was first hospitalized following five outpatient sessions, with the goals of clarifying the diagnosis, optimizing pharmacotherapy, and addressing prolonged school absence. She chose to end the hospitalization after one week, and care continued in the outpatient setting. A total of 13 follow-up sessions were conducted, integrating AIT, psychoeducation, and pharmacological adjustments.

Despite this structured approach, she missed the 14th scheduled session and soon after presented to the emergency department in an acutely suicidal state. This led to a second brief hospitalization. She remains in outpatient care.

Over the course of treatment, clinician-assessed outcomes showed modest improvement, particularly in anxiety and derealization symptoms. Her daily episodes of detachment and memory gaps gradually eased with treatment, improving alongside her ability to manage emotions. By the later stages, dissociation was rare and no longer disruptive, showing that while it had been a major challenge, it responded well to combined medication and therapy. However, core features of BPD and ASD – such as affective instability, compulsive behaviors, and social difficulties – persisted, consistent with the chronic nature of both disorders. Treatment remains flexible and responsive to clinical needs, with focus on crisis prevention, emotional regulation, and improving social functioning.

## 4 Discussion

### 4.1 Challenges in psychiatric diagnosis

A key challenge in this case was the overlap of symptoms across multiple conditions, including BPD, ASD, anxiety disorders, and trauma-related symptoms. Emotional dysregulation, impulsivity, and social communication difficulties complicated the differential diagnosis, particularly in a female patient, where ASD often presents differently than in males (54). Additionally, trauma-related symptoms such as emotional instability and hypervigilance can mimic features of personality disorders, further complicating the diagnostic process (21). Chronic childhood trauma—particularly emotional neglect, physical abuse, and sexual abuse—is a well-established etiological factor in the development of BPD, with lasting effects on emotional regulation, neurobiology, and interpersonal functioning. Compared to other personality disorders, individuals with BPD more frequently report histories of early trauma, which contributes to more severe, comorbid, and treatment-resistant clinical presentations (22). Dissociation represented both a diagnostic and therapeutic challenge in this case, as its severity raised the question of dissociative identity phenomena in the context of complex trauma, even though DID criteria were not ultimately met (45).

Structured assessments like the ADOS-2 were helpful in identifying ASD traits, but they may not fully capture the subtler social difficulties seen in females on the spectrum (55). The developmental anamnesis was challenging to obtain due to family conflict and poor relationships. Self-report measures were also problematic, as emotional dysregulation can influence responses, complicating result interpretation (56). Furthermore, psychosocial stressors, including family instability and an unstable living situation, exacerbated her symptoms, making it difficult to isolate the underlying psychiatric conditions. This case highlights the need for a comprehensive, longitudinal approach to diagnosis, requiring multiple assessments and clinical observations to refine the understanding of the patient’s condition (57).

### 4.2 Strengths and limitations of the approach to the case

This case was managed with a multi-layered approach combining pharmacological treatment, tailored psychotherapy, and psychoeducation. A strength was the integration of AIT for BPD with ASD-specific psychoeducation, addressing identity instability and social-emotional challenges concurrently. The multidisciplinary team ensured a comprehensive understanding of the patient’s needs and supported her autonomy in treatment decisions.

Brexpiprazole was chosen for its receptor profile and tolerability, effectively addressing mood instability, dissociation, and anxiety. Despite limited adolescent-specific literature on its use in BPD, it was well-tolerated, making it suitable for this complex presentation.

Long-term outpatient care was a significant strength, allowing for gradual symptom improvement and emotional regulation through pharmacotherapy and psychotherapy, addressing core challenges of both BPD and ASD.

However, coordination between AIT and psychoeducation was challenging and could be further optimized to better address identity and social-communication difficulties. Diagnostic challenges arose from the incomplete developmental history, complicated by family conflict and mood-dependent variability in self-reports.

Family involvement was inconsistent, as the patient alternated between her grandmother’s and mother’s care, hindering stable family support. Additionally, while crisis management was reactive, earlier crisis planning could have reduced the need for emergency hospitalization.

Despite these limitations, the flexible, integrated approach led to measurable progress, with potential for refinement in similar cases.

### 4.3 Discussion of the relevant medical literature

The diagnostic process followed a comprehensive, multidisciplinary approach, aligning with best practices for complex psychiatric cases, using structured interviews, standardized diagnostic tools (like ADOS-2 for ASD and DSM-5 criteria), and collaboration across disciplines (58). This standard procedure ensured thoroughness in diagnosis.

However, the symptom overlap between BPD and ASD poses challenges, especially in females, where subtle ASD traits may be masked by mood instability and interpersonal difficulties (54). Studies show that females with ASD are often misdiagnosed with personality disorders due to their more subtle social deficits (55).

This case required a holistic approach, integrating findings from multiple sources to address the interplay of social, cognitive, and emotional symptoms. This approach, while time-consuming, is crucial in co-occurring conditions, as patients with both BPD and ASD face heightened social and emotional challenges (13). Additionally, careful differentiation between trauma-related symptoms and mood or personality disorders was necessary, as trauma can mimic or worsen other psychiatric conditions (21).

### 4.4 Take-away lessons from the case

Accurate diagnosis is the foundation of effective intervention in adolescents with overlapping features of BPD and ASD. A longitudinal, multi-informant process—using structured tools, clinical observation, and multidisciplinary input—is essential to disentangle shared traits like emotional dysregulation, interpersonal vulnerability, and cognitive rigidity. As diagnostic clarity evolves, treatment must remain flexible and individualized, combining targeted pharmacotherapy with tailored psychotherapeutic and psychoeducational approaches. Sustained progress depends on responsiveness to the shifting clinical picture and close coordination across interventions.

## Patient perspective

With the patients permission we share her unedited perspective: *I have always seen myself as wrong and getting diagnosed has helped my self acceptance and my coping tremendously, the struggles i’ve struggled with for the longest time finally started making sense to me i was constantly seen as strange but nobody ever wanted to explain to me why and i always felt failed by professionals in my life, so having a therapist and a psychiatrist that finally understood me and took their time with me was such a relief, it’s never been easy and it probably never will but therapy and getting diagnosed really helped me feel better about myself and helped me cope with that one aspect of my life i never really understood, finally putting a name to the face of what was happening in my head has made a giant impact on my life and has made a big difference in how i see myself and the world around me.*

## Data Availability

The original contributions presented in the study are included in the article/supplementary material. Further inquiries can be directed to the corresponding author.
